# East and West

**Published:** 1887-10-29

**Authors:** Leslie Keith

**Affiliations:** Author of "The Chilcotes," "Uncle Bob's Niece," etc.


### East and West.

By Leslie Keith,

Author of "The Chilcotes," "Uncle Bob's Niece," etc.

(*Continued from p.* 70.)

### Chapter V.—A Poet in London.

What had a season in London done for David Jardine? "Send, if you choose, a philosopher to London, but on no account send a poet thither," says Heine, whose own sensitive soul finds speech here. "Send a philosopher, and place him at the corner of Cheapside; he will there learn more than out of all the books of the last Leipzig Fair,—but do not send a poet to London! This hard reality of things—this colossal uniformity—this mechanical movement—this sullenness amidst its pleasures of overgrown London, depresses the imagination and rends the heart."

Among his own green hills nature had seemed to say to David, "Come! understand me—interpret me—make others to see me as you see me"; in Bethnal Green this voice is strangled by the common, sordid cares of life; no one has any ear for it, or time to listen to its pleading. The costermonger with his barrow is the poet of the East-end; he is the lyrist who has the biggest audience. What care we for your babble of hills and streams, of dawns and sunsets? Give us food for our hungry stomachs—we have an appetite that is never satisfied—a craving that knows of no rest; we fight and struggle daily for a morsel of bread—what care we for your mental food? David's disillusionment was, however, a work of time, and though the dull greyness of the streets did not inspire him as he had hoped, he had still his poems—those pastorals that were to bring him fame. He duly set out with a packet of them, carefully rolled and tied, on the Monday morning that followed his arrival. Mary took the utmost pains with the outside of her poet; she brushed his coat, she re-tied his tie; she pushed back the hair from his broad, square brow. She kissed him fondly as a mother might, and could scarce follow him as he went down the dingy street, for a dimness that obscured her sight.

He went with an eager, assured step, the tread of a conqueror. He was a bonnie lad, slim and slight of build, without much physical or mental force; too sensitive to be the hero he dreamed. With the bucolic person of Miss Frankland's imagination he had nothing in common; as a farmer he was a fraud; as a shepherd he must have allowed his sheep a fatal latitude while he communed with the stars; as a poet he was now to be put to the test. He had with him a list of the publishing houses that were likely to vie with each other in the purchase of his wares; he had studied every turn and twist of the way that leads to the modern Grub Street too accurately to be afraid of wandering, and in due time he found himself in the narrow Row where genius is put up to auction.

Editors are notoriously lacking in sensibility; indeed, a heart is no part of their stock-in-trade, and they are generally careful to leave it at home when they go forth in the morning to sit in the chair of the critic. The first editor to whom David made his appeal sent down word that he could not see Mr. Jardine without an appointment. The message was delivered through a speaking-tube, which lent it an additional sting. It was at first sight a repulse, but on consideration there was hope in it, and comfort for a wounded spirit. There was dignity in the sound of an appointment, and the poet resolved to write and make one on his return,—unless, as might very well happen, he had a satisfactory offer in the meantime. The next editor was even more curt. There is, perhaps, only one real poet among a million aspirants to the name, and probably the great man who advised Messrs. White and Black was safe enough in determining that the *rara avis* had not come to his door that summer morning. The world can put up with bad novels, but it does not want indifferent verses, and this young man's were sure to be indifferent, because he was entirely unknown. This was not a very logical way of putting the matter, since the most famous and brilliant writers were necessarily unknown to begin with; but it is an editorial way, as David found. Nobody would take the risk of launching him on an unknown sea. To follow him in his day's adventure would but be to record a long disaster. Some were kind, but their kindness seemed more cruel because they only gave him praise, not hope. The verses had a certain grace and prettiness, but that sort of thing hardly took nowadays. One wanted something more piquant, with more dash and go. Mr. Jardine did not perhaps care to write *vers de société?* The question seemed rather superfluous when one looked at Mr. Jardine. He might try the magazines: they often wanted little things to make up. Thus the book-men basely shuffled him over to their brethren of the serials, and the serial people in their turn blandly suggested that perhaps—"a little volume —— ?"

He looked so pale and spent when he got home, that Mary, who had watched all day at the window, ran out in alarm. Her eyes hungrily sought to read victory in his, and the boy, seeing her, rallied. His eyes brightened, and he held up his head defiantly.

"Not yet," he said. "I am going to write and make an appointment. I find that is the way; they don't care about seeing you casually; they can't give you proper attention."

"Come and have some tea, dear," said Mary gently.

"Tea!" he echoed irritably, "why do you always trouble me with your fussing cares? I tell you I have important letters to write."

"Afterwards, David; you must eat first."

He yielded, but he sat late to write and to read over and over again the precious manuscripts he was about to launch on the world, too feverish and excited to sleep. The next day he wandered out to try his fortune once more; he had a smaller stock of specimens with him this time, since he had sent several of his best poems forth on that melancholy voyage where they would doubtless touch at many ports, and yet perhaps never find a haven. There are a large number of publishers in London, and while there was one left untried the balance of hope was on his side.

So the days went by while he fought his battle, and every dawn found him more eager, more irritable; thinner, too, and shabbier; his boots grew worn with much pacing of the streets, his coat began to show white at the seams, his cheeks had hollows in them, but his eyes were lustrous. Mary looked on with a heart that bled; she was endlessly patient with him; she never reproached him, nor upbraided him for his crossness, his absorption, his indifference over the anxieties that weighed on her, but she suffered as only good women suffer for those they love.

Perhaps she ought not to have pitied him so much, for poets have one immense consolation which other men are denied: they believe in themselves, and when they do not meet with a response from the world, they have the satisfaction of blaming the big public's stupidity.

David was not left without warnings. Mrs. Carter, who looked upon her lodger as a kind of harmless idiot, called him to her one morning, when she had received and was unpacking her week's supply of butter. "This will be something in your line, Mr. David," she said, presenting him with a greasy sheet of paper.

He took it mechanically. There were lines on it, irregular lines, defaced and stained, but recognisable still as poetry.

"Where did you get it?" he asked, with a fine assumption of indifference.

"Why, round the butter, to be sure. It comes so every week. I've had it at times myself to tie up cheese and ham in for the customers; it comes cheaper than paper without the print, though you would hardly think it. If I were you, Mr. David"—she looked at him sharply—"I would take to the grocery line. Folks must eat, but they can take their butter without the poetry. That's my notion, any way."

Was there ever advice more likely to disagree with a poet? A grocer, indeed! David went off with a high head, and yet his heart was heavy within him. The world seemed at least able to exist without his verses; his spirit was growing bitter against this world—this cold, indifferent, careless world, that takes so little heed of hearts that are breaking with despair. There were other sad hearts, there were other strugglers, many of them within the compass of the little street, but he had no eyes for them. It was Mary who saw and understood. She had a deeper sympathy with those other toilers now, because she began herself to know what care meant—care that might one day begin to be want.

She had never known that before. On a farm there is always abundance of food at the least—enough, and to spare—a rough plenty that knows no lack; here in London, where you can get nothing—at all events in the East-end—unless you pay for it (doubtless in Mayfair you can live most handsomely on credit), she began to recognise the value of money. The little store brought from the old home was melting, growing rapidly smaller. When it came to an end—what then?

Before that happened, she went one day to Mrs. Carter.

"We can't afford to live with you any longer," she said. "We must go to some cheaper place for a little—till David succeeds."

Mrs. Carter was very unwilling to let Mary go. She cared nothing for David—he might leave if he would, but not Mary. Mary had proved herself useful almost as a daughter. She was handy in the shop, always busy, always quiet and gentle; she was quick, too, with her needle, and had proved herself skilled in the manufacture of smart caps for the widow's adornment.

"You might stay," she said; "indeed, I would keep you for nothing for the help you could give—I might even spare you a trifle by way of wages—but as for your brother—"

"I will never leave David," said Mary, refusing this generous offer with great promptitude. "There are just the two of us—we can't part."

"And what will you do? for he'll never put bread in your mouth if he should live to be the age of Methuselah."

"I will take in sewing. I can do that; you have said so yourself."

"Do you know how girls who sew are paid?"

"It will help," said Mary bravely. "We have a little yet; enough for a time—enough till David sells his book."

"Well," said Mrs. Carter dryly, "I'm glad to hear it, I'm sure. You must have more money than most of us, if you're going to wait for that day. There's nobody here that can afford to buy poetry, even if it was to get printed in a book; we can scarcely scrape enough together to keep body and soul in life. There's nothing to waste on books, that nobody can make head or tail of when you do set yourself to read them."

"I know that," said Mary gently, "but London is a great world, and there are rich people in it who will listen to David."

The girl's loyalty remained unshaken, but Mrs. Carter, who had not the sister's love to blind her, knew that all the poetry that David would ever write, would be poetry that came to the butterman at last.

Mary took two small rooms at the top of a large tenement in a street that was a great deal dingier than Lavender Lane, because the houses were so much taller that they shut out far more of the sky. In the West-end there are dull streets too, though the houses in them are called mansions: but then the dulness matters less, because it is all on the outside, and only affects the passer-by.

In Bethnal Green this is not so. The sadness that is without is more than matched by the sadness within. You do not pass from the monotony and uniformity of the street to a palace where wealth, and taste, and luxury make you speedily forget the melancholy of our English architecture, and the austerity of our English climate. You pass to interiors where poverty and want allow nothing lovely to dwell, where damp and dirt have their homes, where disorder reigns, where hope never enters. This desolating sadness without and within is the first thing that strikes a stranger in this strange land; it is a reflex of the lives that are lived there—the fights that are fought there, the despair that overwhelms. Death were almost better than life under such conditions, we say who come from happier regions, where perhaps, after some blundering fashion, we worship Art and Beauty. What is there of artistic, of beautiful here, where our brothers and sisters live and die? A flower, perhaps, cherished at some high window—a strip of the sky which shows blue even here on a sunny day, and is bright at nightfall with its little lot of stars.

In Baron Street Mary and David took up their quarters, where they shared in every respect the life of their neighbours, except that they loved order and cleanliness, and found these virtues possible, though no one else dreamt of practising them.

On Mary it fell to make and keep the new home cheerful, and she did it with a brave heart. David took the change philosophically, and without much comment. He was glad to see the last of Mrs. Carter, whose little parable had wounded his pride. He could plan, and brood, and dream even better in the high room in Baron Street, where the atmosphere was not so subtly charged with the odour of ham and cheese. After all, a poet does not ask very much. A table at the window where the light was favourable, his pens and papers in order, a freedom from question or comment, and from the vulgar necessity of knowing anything about a meal until the table was spread,—these were the conditions David asked and obtained.

He shut all knowledge of the world without from him with an ease that is natural to the poetic temperament. A rhyme, a cadence, a tag to a couplet—these absorbed him, and the dream of coming fame consoled him. The praise of men was in his ears, and compared with it the groans and sighs of the fighting mass of humanity round him were dim as an echo.

It was left to Mary to see, to hear, to feel, to suffer an ache that would never cease. And all the while she watched David narrowly, closely, with the jealous eyes of love. The summer days in that close air were telling on him, the days of deferred hope. She still believed in his ultimate victory. He must succeed—but suppose success came too late? Her heart stood still at the bare thought.

That very afternoon, when David was busy and would not miss her, she put on her bonnet and slipped out to post with her own hand the schoolmaster's long-delayed letter.

"For David's sake," she whispered to herself. Before David's need, even pride must make surrender.

(*To be continued.*)

